# Books and Magazines

**Published:** 1907-04

**Authors:** 


### Books and Magazines.

A Manual of Obstetrics.—By A. F. A. King, M. D., Professor of Obstetrics and Diseases of Women in the Medical Department of the George Washington University, Washington, D. C., and in the Medical Department of the University of Vermont, etc. Tenth edition, enlarged and thoroughly revised. 12mo., 688 pages, with 30 illustrations and three colored plates. Cloth, $2.75, net. Lea Brothers & Co., Philadelphia and New York, 1907.

*King* is one of the *perennial* books. It is now beginning its second quarter-century with its tenth edition and has thus spanned with vigor the most active and exacting period in medical history. No other obstetrical book extant has such a record. Every fact has a reason, and the ever-growing favor bestowed on *King* can have but one basis, namely merit. The author combines the faculties of a teacher and practitioner, and accordingly has been able to select what is important and to present it clearly. The student thus easily acquires a grasp of everything essential, and the accoucheur can turn to these pages for reference on any point of practice. Suiting both classes of readers this single handy volume receives their combined demand and hence goes through successive editions, enabling the author always to keep it revised to date, as he has just done again, with considerable enlargement both in text and engravings, and with the addition of colored plates.

---

Text-Book of Psychiatry.—A Psychological Study of Insanity for Practitioners and Students. Br. Dr. E. Mendel, A. O., Professor in the University of Berlin. Authorized Translation. Edited and enlarged by William C. Krauss, M. D., Buffalo, N. Y., President Board of Managers Buffalo State Hospital for Insane; Medical Superintendent Providence Retreat for Insane; Neurologist to Buffalo General, Erie County, German, Emergency Hospitals, etc.; Member of the American Neurological Association. 311 pages. Crown Octavo. Extra Cloth. $2.00, net. F. A. Davis Company, Publishers, 1914-16 Cherry street, Philadelphia, Pa.

For many years Prof. Mendel has been in the front rank of German men of science. His work is well known in Europe, but this, I believe, is the first English translation. In most large cities there are psychiatric clinics, attendance upon which is obligatory on students, and an examination upon psychiatry is required for license to practice in most States. Hence this translation fills an evident want and is most timely.

---

Psychology Applied to Medicine.—Introductory studies by David W. Wells, M. D., lecturer on Mental Physiology, and Assistant in Ophthalmology, Boston University Medical School; Ophthalmic Surgeon, Massachusetts Homeopathic Hospital, Boston; Oculist, Newton (Mass.) Hospital. Illustrated, nearly 200 pages, with Bibliography and Index. 12mo. Extra quality of paper. Neatly bound in cloth. Price, $1.50, net. F. A. Davis Company, Medical Publishers, 1914-16 Cherry street, Philadelphia, Pa.

The present essay has developed as a result of several years' lecturing to medical students, and is based on a practical knowledge of their needs.

The leading features of the book are:

1. A clear statement of the important facts of medical psychology, such as Reason and Instinct, Habit, the Subconscious, the Evolution of the Special Senses, and the elucidation of many practical problems of the Sense of Sight, among which is a detailed consideration of the Inverted Retinal Image. This material occupies the first few chapters.

2. Hypnotism (its history, methods of induction, and theories concerning it) is treated in three chapters. This is a valuable resume of the present status of the subject, together with the account of considerable original experimentation. Its value and place in the practice of medicine are carefully considered.

3. The great subject of mental healing in its many forms occupies the three remaining chapters. An attempt is made to find the underlying therapeutic principle, which is so generally obscured by the false notions and extravagant claims of the various sects.

The book concludes with a critical examination of the prevalence of a psychic element in all forms of modern medical methods.

A book of 1000 pages might easily have been made of the material presented, but such "padding" would have spoiled the author's avowed purpose, namely, to present a readable and trustworthy introduction to the subject.

---

THE NEW HYGIENE.—By Elie Metchnikoff, author of *The Nature of Man,* with preface by E. Ray Lankester. Chicago, 1906. W. T. Keener & Co., publishers. Cloth, boards, $1.00, net.

This little book is made up of Metchinkoff's celebrated Harben Lectures on The New Hygiene, or The Prevention of Infectious Diseases, translated by the no less famous Professor Lankester. It is a gem. 1. The Hygiene of the Tissues. 2. The Hygiene of the Alimentary Canal. 3. Hygienic Measures Against Syphilis. No physician should be in ignorance of what is here taught.

A Compend on Bacteriology, Including Animal Parasites.—By Robert S. Pitfield, M. D., Pathologist to Germantown Hospital; also Pathologist to the Hospital for Lung Diseases, Chestnut Hill; Pathologist to the Widener Memorial School; late Demonstrator of Bacteriology at Medico-Chirurgical College, Philadelphia. Four plates and 80 other illustrations. Philadelphia, 1907. P. Blakiston's Son & Co. $1.00, net.

The nature and purpose as well as the scope of this book are sufficiently set forth in the title. Medical students especially will "need it in their business" when preparing for "exams."

---

Manual of Chemical Chemistry.—By A. E. Austin, A. B., M. D., Professor Medical Chemistry and Toxicology in the Medical Department of Tuft's College, Boston. D. C. Heath & Co., publishers, Boston, 1907. Price, $1.75.

This is a new kind of chemistry, designed, the author states, "to make chemistry the handmaid of clinical medicine." The book is admirably adapted to the purpose stated.

---

Thornton's Pocket Medical Formulary. — New (8th) edition, revised to accord with the new U. S. Pharmacopœia. Containing about 2000 prescriptions with indications for their use. In one leather bound volume. Price, $1.50, net. Lea Brothers & Co., Publishers, Philadelphia and New York, 1907.

It would be difficult to mention a more frequently useful work than *Thornton's Formulary*. The author is peculiarly qualified to render such a service, as he unites in himself a knowledge of the three necessary branches, being a graduate in pharmacy, a professor of materia-medica in a leading medical college, and an active practitioner of many years' standing. He has here presented the collective experience of his profession as to the best measures for combating disease. He has arranged *Diseases* alphabetically, and under each has given the best formulæ for simple cases, as well as for the various stages and complications, with quantities both in the ordinary and metric systems. A feature peculiar to this work, and one of obvious value, is found in the *Indications* and annotations for a choice between the various formulæ according to the conditons to be met. Critical study has been given to each formula in all its parts, as well as to palatability and compatibility. No point desirable in such a work has been overlooked. The most experienced physician will find it useful as a reminder, and his younger confrere will perform his duty better both to his patient and himself with its suggestions at hand for quick reference. That practitioners widely appreciate its value is shown by the frequent demand for new editions, a point of special importance in a work dealing with so rapidly advancing a department as Therapy. In each of its eight editions, the author has embodied the latest and best information, so that the profession may consult this hand-book with confidence in finding it always up to date. This is particularly important in the case of this new edition, as it has been revised to accord with the new United States Pharmacopœia, in which many official changes have been made in the strength of drugs and their preparations. An undesirable *risk* now attends the use of all medical books dealing with drugs according to the old and obsolete Pharmacopœia. It is obviously essential that doctor and druggist should both follow the new and legal standard. The transition from the old to the new is facilitated by Dr. Thornton's book.

---

Here's Appleton's latest publication:

DISEASES OF THE LUNGS.—A practical presentation of the subject for the use of students and practitioners of medicine. By Robert H. Babcock, A. M., M. D., author of Diseases of the Heart and Arterial System; late Professor Clinical Medicine and Diseases of the Chest, College Physicians and Surgeons (medical department) Illinois State University, Chicago, etc., etc. 12 colored plates and 104 text illustrations. First edition. New York and London. D. Appleton & Co., 1907. Cloth, $6.00, net.

This work is a companion volume to that upon *Diseases of the Heart,* published a short time ago. We believe this to be the most exhaustive and yet comprehensive work upon this subject which has ever been published. The author's individuality is shown throughout the work. His style is attractive.

Dr. Babcock has in this work, as in his book upon *Diseases of the Heart,* given many records of interesting cases which will be of great interest and very helpful in diagnosis for the general practitioner.

*Differential Diagnosis and Treatment* are given the attention which their importance demands. Every physician in the land will be benefited by having this work for consultation. No other book in the English language so practically and exhaustively treats these important diseases.

---

PLASTER OF PARIS AND HOW TO USE IT.—By Martin W. Ware, M. D., Adjunct Attending Surgeon, Mount Sinai Hospital; Surgeon to the Good Samaritan Dispensary; Instructor in Surgery, N. Y. Post Graduate Medical School. 12mo; 72 illustrations, about 100 pages. Surgery Publishing Co., 92 William street, New York City. Cloth, $1.00.

This is one of the most useful books ever presented, not only on account of the general demand for the information and instructions upon the subject which this book so explicitly, practically and comprehensively covers, but because this knowledge was not previously available except from such a vast experience as enjoyed by Dr. Ware, or, in part, by reference to many books on allied subjects.

It is a vivid narrative, profusely illustrated, of the many uses to which plaster of paris is adaptable in surgery. The whole subject, from the making of the bandage to its use as a support in every form of splint, corset or dressing, is graphically described and illustrated. The use of plaster of paris in dental surgery is also covered. The book is presented in the artistic manner characteristic of the productions of the Surgery Publishing Company. It is printed upon coated book paper and attractively bound in heavy red buckrum, stamped in white leaf and gold. Price, $1.00.

